# Progress in Neurology

**Published:** 1901-04-27

**Authors:** 


### Progress in Neurology.

**Tremor.**—Williamson[1] has lately discussed the clinical characters of various forms of tremor, and described some of the rarer forms. The relation of the tremor to voluntary movement enables us to arrange the cases into three groups: (1) Tremor occurring during repose, but ceasing or diminishing on voluntary movement with attention, *e.g.* tremor in paralysis agitans; (2) Tremor occurring only on voluntary movement and ceasing during repose, *e.g.* disseminated sclerosis; (3) Tremor which occurs during repose, but is much greater during voluntary movement, as in most cases of alcoholic, senile, asthenic, simple and hysterical tremor. Hereditary tremor is occasionally observed—it occurs in families, and is the only abnormal symptom. The tremor may affect various voluntary muscles, and occasionally all the muscles, but is most frequently met with in the arms, less frequently in the legs, trunk, neck, lower jaw, and tongue. The type of the tremor is different in different families; in some cases it is a coarse intention tremor, not very frequent, of almost convulsive character, and eventually complicated by convulsive contractions in single groups of muscles. In other cases it is a fine rhythmical oscillating tremor which can be checked by voluntary effort. Tremor may be associated with gross lesions of the brain, *e.g.* hemiplegia, tumours of the optic thalamus, crus, pons, corpora quadrigemina, and cerebellum. Cases are occasionally met with in which tremor has been present since early infancy. Thus, in a boy, aged 13, there was a fine tremor of the hands, which was stated to have been present since birth. It occurred on voluntary movement but ceased when the arm was supported. There were no symptoms of disseminated sclerosis and the tremor was finer and less jerky than in that disease. There is another ill-defined group of cases, in which at present no cause can be made out. Tremor is the only symptom or the only important symptom; it persists for years, and apart from the inconvenience it causes there is no other complaint. These cases do not develop into paralysis agitans or disseminated sclerosis. The tremor may be fine or coarse; it is often increased by movement or mental emotions; during repose in some cases it diminishes or ceases, but in other cases it continues. The hands are the parts most affected. A very rare disease which is a cause of tremor is myokymia—a condition in which there is a persistent and diffused quivering of the muscles.

**Treatment of Epilepsy.**—Pierce Clark,[2] of the Craig epileptic colony, summarises as follows the present state of surgical and medical treatment of epilepsy. Idiopathic epileptics with typical grand-mal seizures should never be trephined. Idiopathics in whom seizures are of the Jacksonian type should be trephined only when infantile cerebral palsies can be excluded, and when the family and personal degeneracy is at a minimum; if operation is determined upon in such cases, a very thorough removal of the epileptogenic area should be made—even then but a fraction of 1 per cent. recover from their epilepsy. Traumatic epileptics may be trephined when the injury is definitely proven, and has existed not more than two years. The prognosis will then largely rest on the degree of neurotic pre-disposition present. The earlier the trephining is resorted to after the convulsions begin the better the prognosis. All epileptics trephined for whatever cause must be given post-operative bromide treatment for years. The medical treatment which gives the best results is as follows: a combination of diet, regular occupation and personal hygiene, with the administration of bromides, which, singly or combined, still remain our chief sedative for the epileptic state. The bromides to be effective in chronic and long-standing cases must be given in large daily doses to suppress convulsions. They should be given gradually to find the sedative level, and at this level it is one's duty to maintain them with physical and mental comfort to his patient. Hot and cold baths, alimentary antisepsis and massage are absolutely essential to successful bromide medication. Salt starvation or semi-salt starvation is a great adjuvant to the bromide treatment, and should be thoroughly tried in all cases in which bromides are apparently contraindicated before they are discarded. The rationale of salt starvation is the recently discovered fact that bromine can substitute chlorine in the tissues. Hence if salt be withheld bromides are more greedily absorbed by the tissues. It is found that by this method the usual amount of bromides can be reduced one-half, and still the same therapeutic result be obtained.

**Disease of the Prefrontal Lobes.**—Churton[3] has recorded some cases of lesions of the prefrontal lobes. The function of these lobes is briefly:—(1) To permit or to inhibit certain proposed acts, according to the memory of former results of such acts; and (2) to excite the lower centres of sense and motion in the absence of external stimuli. Diseases of the lobes can be divided into irritation disorders and depression disorders. Cases were related of (1) simple automatism due to bilateral abscess in the prefrontal region; (2) curious acts and mistimed humour, due to sarcoma of the third right frontal gyrus; (3) whimsical conduct during life—due to gummata in the prefrontal lobes; (4) a man, aged twenty-seven years, with humerous conduct and anosmia in the right nostril —due to aneurysm of the anterior communicating artery invading the right lobe; (5) a man, aged thirty-six years, who in June 1899 could not smell flowers, in February 1900 he was sleepy, blundered in his work, was forgetful, and negligent about dress. In April he lost interest in home and family. In June, urine and fæces were passed unconsciously and unnoticed. He died in September, when a psammo-sarcoma as large as an orange was found growing from the falx cerebri and compressing both prefrontal lobes. It is not the absence of intelligent replies and other actions in response to excitations from without, but the absence of spontaneity—*i.e.* of excitation from within—which is the distinctive sign of prefrontal paralysis.

**Subacute, Combined Degeneration of the Spinal Cord.**—Batten, Collier, and Russell[4] have given an account of a recently recognised affection of the nervous system, which presents a distinctive clinical picture and an equally distinctive morbid anatomy, based on seven cases confirmed by autopsy. The average age of the patients was forty years. In several a definite history of syphilis was obtained. Though anæmia was present in some of the cases, it was always of the secondary variety, and was absent in some otherwise typical cases. The clinical picture, except in its earliest stages, is very definite, and allows of three stages being recognised:—(1) Slight spastic paraplegia with slight ataxy; (2) Severe spastic paraplegia with marked anæsthesia of the legs and trunk; (3) Complete flaccid paraplegia with absent knee-jerks. The first stage usually occupied one-half or threequarters of the whole duration of the illness, whether this was chronic or acute. The transition from the first to the second stage was abrupt, and was marked by inability to walk or stand; the duration of this second stage was on the average about five weeks. The transition from the stage of spastic paraplegia to the third stage, characterised by flaccid paraplegia, occurred rapidly in the course of a few days and was always associated with pyrexia. This last stage lasted as a rule about six weeks, death resulting from sudden syncope or respiratory failure. One patient who appeared to be the subject of this affection recovered, and is now well; but with this exception there is nothing to justify any but a hopeless prognosis, and the duration of life is usually short. No treatment influences the nervous manifestations. The essential morbid condition is an intense degeneration of the whole matter of the spinal cord, affecting the posterior, lateral, and anterior columns, and indeed in the midthoracic region of the cord, where the disease is most pronounced, the whole of the white matter is destroyed except that immediately bounding the grey matter. The grey matter and the peripheral nerves are practically normal.

**Kernig's Sign.**—Roglet[5] has recently made an important contribution on the diagnostic value of Kernig's sign in cerebral affections. Kernig pointed out that in meningitis the knee cannot be fully extended if the thigh be placed at right angles to the trunk. The cause of this is a contracture of the flexors of the knee. If, however, the thigh be in the same straight line with the trunk (as in lying flat in bed) the knee can be extended. Roglet finds that this sign is not exclusively present in meningitis and may be met with in other forms of cerebral irritation. In 85 per cent. of cases of meningitis, whether due to meningococci, pneumococci, streptococci, or staphylococci, it was easily elicited. In tuberculous meningitis it was somewhat less frequent. It makes its appearance when the disease is well established, and may vary in intensity from day to day. Spinal meningitis need not be present co-extensively with cerebral to give rise to the sign.

**Clinical Forms of Spinal Syphilis.**—Williamson[6] has recently given a useful summary of the various clinical forms of spinal syphilis. (1) Meningomyelitis is the commonest. There are at first meningeal symptoms only (pain in the back and indications of implication of spinal nerve roots) followed in course of time by symptoms of involvement of the spinal cord, which consist of paraparesis or paraplegia usually with increased tendon jerks and rigidity and frequently with bladder symptoms. There may be partial or complete anæsthesia to all forms of sensation, sometimes loss of sensation to temperature (especially to cold) is the chief or only sensory disturbance. (2) Chronic meningitis, without indications of involvement of the spinal cord, is very rare. (3) Acute syphilitic paraplegia: the symptoms resembling those of acute transverse myelitis. The phenomena in these cases are practically the same as in acute transverse myelitis from other cause. (4) Erb's syphilitic spinal paralysis is a distinct clinical variety. There is spastic paresis or paralysis with increased tendon jerks and rigidity and affection of the bladder, with only slight, though distinct, affection of sensation. The onset of the disease is gradual. Marked paresis gradually develops and only exceptionally is there complete paraplegia. The upper half of the body is unaffected. (5) In rare cases of spinal syphilis, the symptoms have been those of a localised meningeal or intramedullary spinal tumour, and a gumma has been diagnosed. The diagnosis of spinal syphilis is of great importance, especially at an early stage when good results may be expected from prompt treatment. The general indications in favour of the nature of a syphilitic lesion are:—The history of previous syphilitic infection, signs of present or past syphilis on the body, the presence of associated cerebral symptoms, the relatively slight intensity of the cord disease as compared with the extensive area involved, multiplicity of lesions. Many authors attach much importance to pain in the back, which is worse at night. The prognosis is on the whole better than in other affections of the spinal cord; it is better when the meninges are involved than when there are signs of involvement of the substance of the cord. The treatment should be with mercury and iodide, and most thorough.

[1] Williamson, Med. Chron., Oct. 1900. [2] Pierce Clark, Med. Rec., Jan. 12, 1901. [3] Churton, Lancet, Jan. 26, 1901. [4] Batten, Collier, and Russell, Brain, 1900. [5] Roglet, Thèse de Paris, 1900. [6] Williamson, Edin. Med. Jour.

